# Usefulness of a novel narrow-diameter endoscope for endoscopic balloon dilation of esophageal strictures

**DOI:** 10.1055/a-2223-4325

**Published:** 2024-01-09

**Authors:** Koichi Soga, Toshikuni Suda, Ikuhiro Kobori, Yasumi Katayama, Masaya Tamano

**Affiliations:** 1 26263 Department of Gastroenterology, Dokkyo Medical University Saitama Medical Center, Koshigaya, Japan

Esophageal stricture is a narrowing of the esophageal lumen, often causing esophageal obstruction 1 2 . Dilation of strictures by endoscopy using balloon dilation is indicated to restore the patency of the esophageal lumen 3 . We present the case of a 55-year-old Japanese man who presented with chest discomfort. Upper gastrointestinal endoscopy (UGE) revealed a full-circumferential ulcer in the mid-portion of the esophagus, without obvious dysmorphic epithelium ( Fig. 1 ). In the absence of obvious malignancy on biopsy, proton pump inhibitors (PPI) were prescribed for severe reflex esophagitis. On day 36 of PPI treatment, the patient reported aggravation of esophageal stasis with solid food. On UGE, an esophageal stricture was observed and was treated by endoscopic esophageal dilatation (EED) using a 12-mm-diameter balloon (CRE PRO GI Wireguided Balloon; Boston Scientific, USA). EED needed to be repeated 2 weeks later. On this second occasion, the stricture was assessed using an ordinary 9.8-mm-diameter upper gastrointestinal endoscope (EG-840T; Fujifilm, Japan), as used on the previous occasion. The endoscope could not be passed through the region of stricture due to scarring in the area of the ulcer ( Fig. 2 ). Therefore, we selected to use a narrow-diameter, 7.9-mm upper gastrointestinal endoscope (EG-840TP; Fujifilm). Although the narrow-diameter endoscope could not be passed through the region of stenosis, it could be safely inserted into the esophageal inlet, providing a more detailed assessment of the stricture compared to the 9.8-mm diameter endoscope. The narrow-diameter endoscope includes a 3.2-mm hole for endoscopic forceps, which allowed us to perform EED using a 13.5-mm-diameter balloon (CRE PRO GI Wireguided Balloon) without difficulty ( Fig. 3 ; Video 1 ). After EED, the endoscope could be passed through the region of stenosis without resistance. Based on our experience, we propose that the novel narrow-diameter endoscope is a potential first-choice endoscope for safe and reliable EED.

**Fig. 1:**
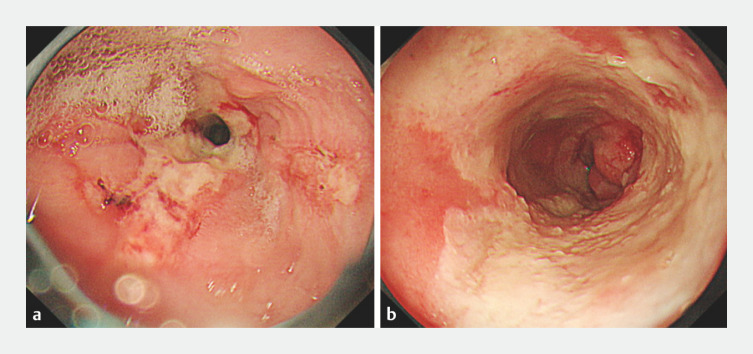
Initial upper gastrointestinal endoscopy showing a full-circumferential ulcer in the mid-portion of the esophagus, with no evidence of obvious dysmorphic epithelium. **a** Longitudinal ulcer observed at the oral edge of the esophageal ulcer. **b** Circumferential esophageal ulcers are visible.

**Fig. 2:**
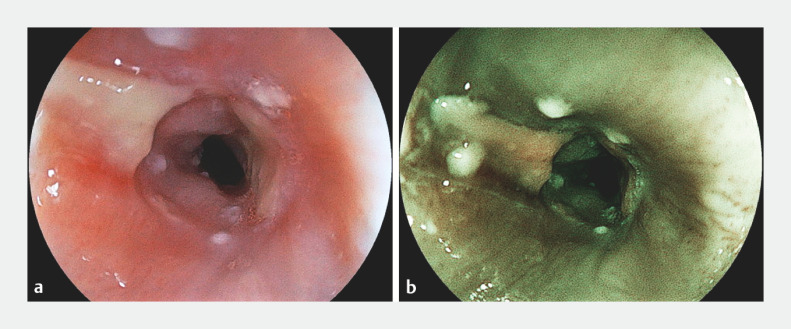
Images of the second endoscopic esophageal dilatation (EED) procedure. A 9.8-mm upper gastrointestinal endoscope was first used to assess the esophageal stricture (EG-840T, 9.8 mm, Fujifilm, Japan). **a** White light imaging; **b** blue laser imaging.

**Fig. 3:**
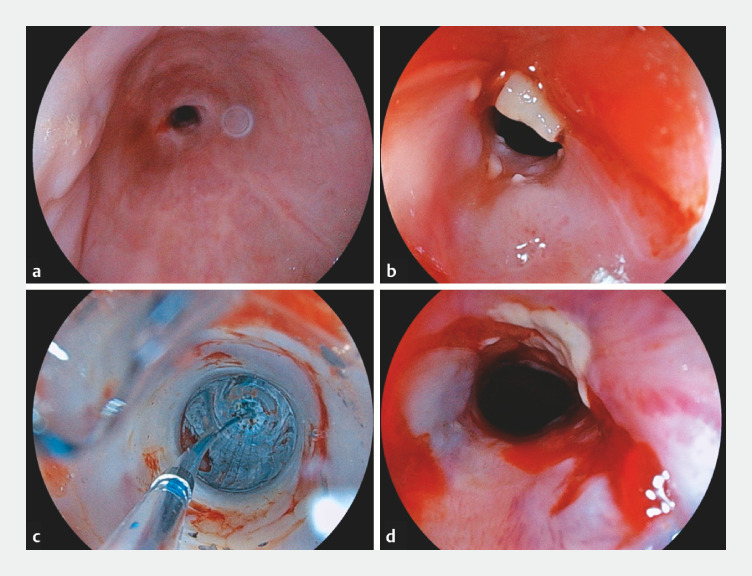
**a, b** The 9.8-mm upper gastrointestinal endoscope could not be passed safely through any region of the stenosis and was therefore exchanged for one of a narrower diameter that could pass through the esophageal inlet in order to further evaluate the stricture. **c, d** EED was subsequently performed without difficulty using the narrow endoscope with a 13.5-mm diameter balloon.

A novel narrow-diameter endoscope was useful for performing endoscopic balloon dilation of an esophageal stricture.Video 1

Endoscopy_UCTN_Code_TTT_1AO_2AH

## Notes

Endoscopy E-Videos https://eref.thieme.de/e-videos
*E-Videos* is an open access online section of the journal *Endoscopy* , reporting on interesting cases and new techniques in gastroenterological endoscopy. All papers include a high-quality video and are published with a Creative Commons CC-BY license. Endoscopy E-Videos qualify for HINARI discounts and waivers and eligibility is automatically checked during the submission process. We grant 100% waivers to articles whose corresponding authors are based in Group A countries and 50% waivers to those who are based in Group B countries as classified by Research4Life (see: https://www.research4life.org/access/eligibility/ ). This section has its own submission website at https://mc.manuscriptcentral.com/e-videos .

## References

[1] Desai JP Moustarah F Esophageal stricture. [Updated 22 May 2023]. In: StatPearls [Internet]. Treasure Island, Florida: StatPearls Publishing; 2023 https://www.ncbi.nlm.nih.gov/books/NBK542209/

[2] Siersema PD Treatment options for esophageal strictures Nat Clin Pract Gastroenterol Hepatol 2008 5 142 152 10.1038/ncpgasthep1053 18250638

[3] Sarma MS Tripathi PR Arora S Corrosive upper gastrointestinal strictures in children: Difficulties and dilemmas World J Clin Pediatr 2021 10 124 136 10.5409/wjcp.v10.i6.124 34868889 PMC8603639

